# The Anti-Melanoma Effect of Betulinic Acid Functionalized Gold Nanoparticles: A Mechanistic In Vitro Approach

**DOI:** 10.3390/ph15111362

**Published:** 2022-11-05

**Authors:** Roxana Ghiulai, Alexandra Mioc, Roxana Racoviceanu, Marius Mioc, Andreea Milan, Alexandra Prodea, Alexandra Semenescu, Cristina Dehelean, Lucian Barbu Tudoran, Ștefana Avram, Cristina Trandafirescu, Codruța Șoica

**Affiliations:** 1 Department of Pharmacology-Pharmacotherapy, Faculty of Pharmacy, Victor Babes University of Medicine and Pharmacy, Eftimie Murgu Square, No. 2, 300041 Timișoara, Romania; 2 Research Centre for Pharmaco-Toxicological Evaluation, Victor Babes University of Medicine and Pharmacy, Eftimie Murgu Square, No. 2, 300041 Timișoara, Romania; 3 Department of Anatomy, Physiology and Pathophysiology, Faculty of Pharmacy, Victor Babes University of Medicine and Pharmacy, 2nd Eftimie Murgu Sq., 300041 Timișoara, Romania; 4 Department of Pharmaceutical Chemistry, Faculty of Pharmacy, Victor Babes University of Medicine and Pharmacy, Eftimie Murgu Square, No. 2, 300041 Timișoara, Romania; 5 Department of Toxicology, Faculty of Pharmacy, Victor Babes University of Medicine and Pharmacy, 2nd Eftimie Murgu Sq., 300041 Timișoara, Romania; 6 Electron Microscopy Laboratory, Faculty of Biology and Geology, “Babes-Bolyai” University, 5–7 Clinicilor Street, 400006 Cluj-Napoca, Romania; 7 Electron Microscopy Integrated Laboratory, National Institute for R&D of Isotopic and Molecular Technologies, 67–103 Donat Street, 400293 Cluj-Napoca, Romania; 8 Deparment of Pharmacognosy, Faculty of Pharmacy, Victor Babes University of Medicine and Pharmacy, 2nd Eftimie Murgu Sq., 300041 Timișoara, Romania

**Keywords:** betulinic acid, gold nanoparticles, melanoma, apoptosis, mitochondria, protein expression, CAM assay

## Abstract

Implementing metallic nanoparticles as research instruments for the transport of therapeutically active compounds remains a fundamentally vital work direction that can still potentially generate novelties in the field of drug formulation development. Gold nanoparticles (GNP) are easily tunable carriers for active phytocompounds like pentacyclic triterpenes. These formulations can boost the bioavailability of a lipophilic structure and, in some instances, can also enhance its therapeutic efficacy. In our work, we proposed a biological in vitro assessment of betulinic acid (BA)-functionalized GNP. BA-GNP were obtained by grafting BA onto previously synthesized citrate-capped GNP through the use of cysteamine as a linker. The nanoformulation was tested in HaCaT human keratinocytes and RPMI-7951 human melanoma cells, revealing selective cytotoxic properties and stronger antiproliferative effects compared to free BA. Further examinations revealed a pro-apoptotic effect, as evidenced by morphological changes in melanoma cells and supported by western blot data showing the downregulation of anti-apoptotic Bcl-2 expression coupled with the upregulation of pro-apoptotic Bax. GNP also significantly inhibited mitochondrial respiration, confirming its mitochondrial-targeted activity.

## 1. Introduction

Natural products have long been recognized as privileged structures in terms of interaction with various protein targets, therefore inspiring the development of modern small drug molecules; thus, more than half of the currently used drugs originate from either natural products or their semisynthetic derivatives [1]. In addition, natural products provide key pharmacophores in the treatment of various pathologies, including cancer; in fact, there are a large number of studies on the potential discovery and use of novel anticancer agents of natural origin. These efforts are supported by the reality of cancer cases nowadays, which, according to the International Agency for Research on Cancer, are expected to grow to 27.5 million by 2040, mainly due to population growth and aging but also to the increased prevalence of risk factors such as poor lifestyle habits [2].

Betulinic acid (3β-hydroxylup-20(29)-en-28- oic acid, BA) is a lupane-type pentacyclic triterpene isolated almost 100 years ago from *Gratiola officinalis* [3] which captured the interest of researchers once its selective anti-melanoma activity was reported by Pisha et al. in 1995 [4]. BA displays a remarkable plethora of biological effects that justify the intense work conducted worldwide in order to identify feasible options for its use as a therapeutic agent; it was the subject of a phase I/II clinical trial in the form of topical application against dysplastic nevi, but the study was discontinued without publishing any results [5]. BA can bind directly to target receptors, while some of the underlying mechanisms of the BA antitumor effects include the activation of mitochondrial pathways resulting in apoptosis, cell cycle regulation and antiangiogenic activity [6]. Of note, BA lacks systemic toxicity while inducing only slight photosensitive effects in experimental mice; therefore, the combination of strong pharmacodynamic properties with a low toxicological profile makes triterpenic acid a very promising alternative as an anticancer agent. However, in order to be able to enter the therapeutic arsenal, BA must meet specific requirements related to bioavailability, which is diminished by the compound’s low water solubility, a common issue for pentacyclic triterpenes [7,8,9]. Different options have been addressed to overcome this obstacle, either through the design of semisynthetic derivatives [3] or through various formulations; the latter approach covers a wide range of possibilities, such as co-crystals [10], cyclodextrin complexation [11], nanoformulations [12], etc. Among the huge range of nanoformulations, metallic nanoparticles (NPs) have attracted a lot of interest due to their multiple advantages, such as small sizes that enable their penetration through biological membranes as well as the possibility of tuning their surfaces in order to achieve optimized pharmacokinetic properties [13].

Gold nanoparticles can be easily synthesized in various shapes and sizes [14] and act as versatile nanocarriers in targeted drug delivery while also exerting intrinsic biological effects, such as antiproliferative, antiangiogenic and proapoptotic [15]. Pentacyclic triterpenes can be loaded at the surface of gold nanoparticles either via a gold-thiolate bond or through hydrophobic or electrostatic interactions [16]. Betulinic acid was conjugated with epigallocatechin gallate-capped gold NPs additionally functionalized in order to specifically target mitochondria; the resulting delivery nanosystems were later assessed in terms of cellular uptake and efficacy in several human cancer cells, revealing three times lower IC_50_ values for the loaded BA compared to the drug alone [17]. Our group previously synthesized two types of betulin-conjugated gold NPs, with or without thiolated polyethylene glycol, which exhibited sizes within 50 and 70 nm, depending on the preparation method and the parameters of surface modulations. All NPs were tested against two melanoma cell lines exerting cytotoxic and apoptotic effects in a time- and dose-dependent manner [7]. Despite these promising results, to the best of our knowledge, the literature contains scarce reports regarding the use of gold NPs as carriers for pentacyclic triterpenes.

The current study focuses on the synthesis of betulinic acid-loaded gold NPs synthesized using cysteamine as a surface linker for the phytocompound, followed by their in vitro assessment in melanoma cells as well as human keratinocytes. The main aim is to unravel potential therapeutic benefits provided by the combination of the triterpenic acid with the metallic nanocarrier but also to decipher the underlying mechanisms in terms of anticancer activity.

## 2. Results

### 2.1. Synthesis of Citrate-Capped GNP and BA-GNP

**Figure 1:**
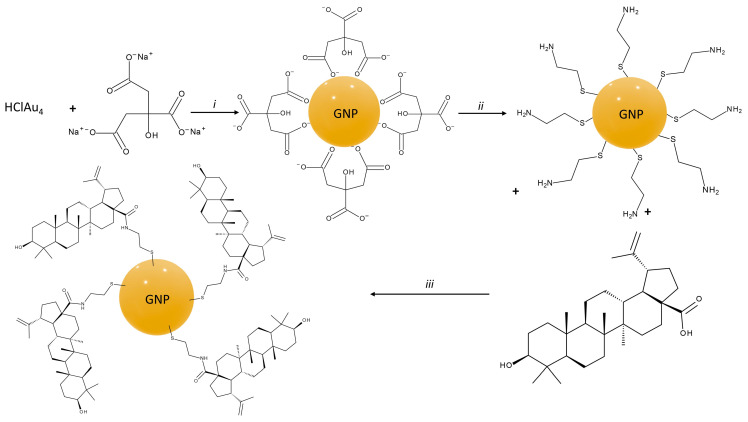
Synthesis route for citrate-capped GNP and BA-GNP; conditions: (*i*) H_2_O, boil, (*ii*) Stirred at r.t. for 10 min; (*iii*) EDC, ultrasonication, 1 h, after which stirred at r.t. for 24 h.

The synthesis of citrate-capped GNP was achieved by the reduction of chloroauric acid with trisodium citrate (Figure 1). The functionalization of GNP with BA was achieved by first attaching cysteamine onto the surface of GNP through the means of gold thiolate bonds. Following this step, the free amino group was used to link the COOH group of BA by carbodiimide-assisted coupling using N-(3-Dimethylaminopropyl)-N′-ethylcarbodiimide hydrochloride (EDC) (Figure 1).

### 2.2. UV-VIS Characterization of GNP and BA-GNP

**Figure 2:**
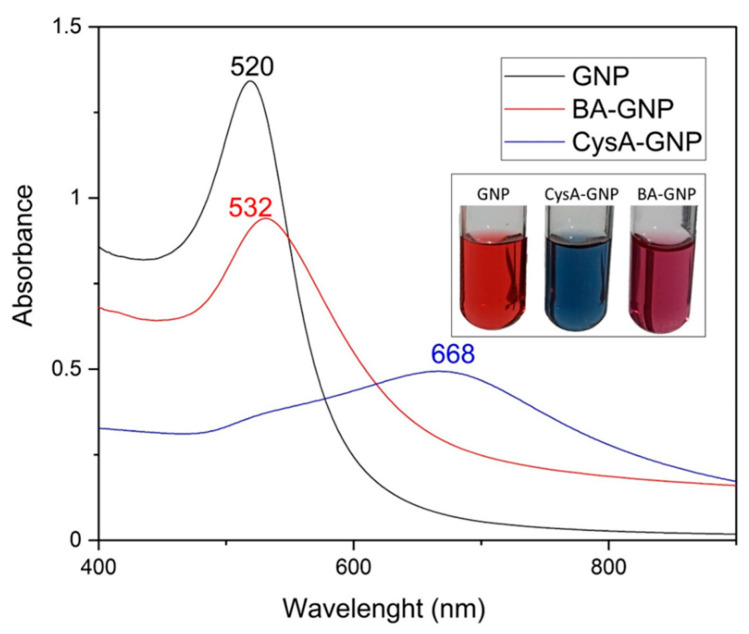
UV-VIS spectra and nanosuspension physical aspect of citrate-capped GNP, BA-GNP and the intermediate formed CysA-GNP.

**Figure 3:**
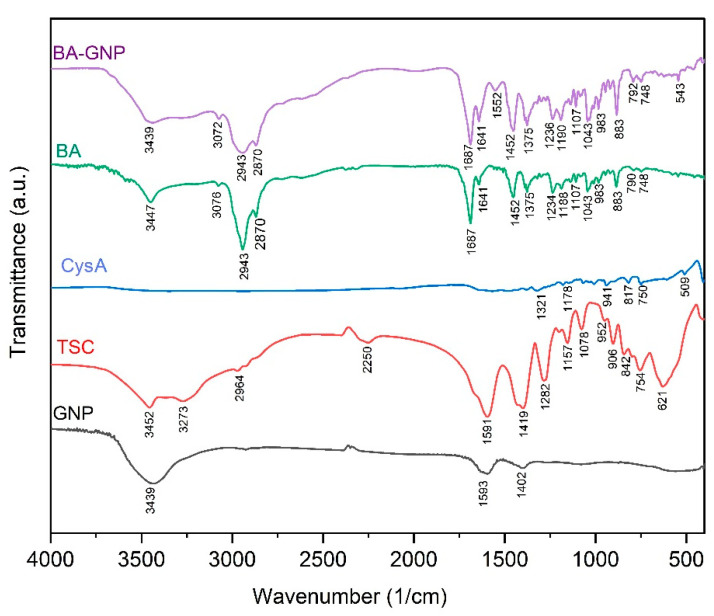
FTIR spectra of BA-GNP, BA, cysteamine (CysA), trisodium citrate (TSC) and citrate-GNP.

GNP exhibits surface plasmon resonance (SPR), a characteristic property that allowed researchers to achieve a deeper understanding of how the size, shape and uniformity of the nanoparticle sample impacts its UV-VIS spectra. Based upon this knowledge, they subsequently developed various methods to demonstrate how changes in the UV-VIS spectra of GNP are directly correlated with changes in nanoparticle shape, size and concentration [18,19]. The formation of GNP by the citrate reduction method was confirmed by UV-VIS spectroscopy. GNP exhibited the characteristic maximum absorbance peak at 520 nm (Figure 2). While cysteamine addition to the GNP suspension instantly produced a rapid color change to blue and a drastic blue shift in the UV-VIS spectra (Figure 2), the BA functionalization of GNP reverted the color of the nanosuspension to purple. Blue shift changes in the SPR maximum of GNP can be correlated with a decrease in overall nanoparticle size, surface functionalization, or an increase in size dispersity [18,19]. The recorded UV-VIS spectra of BA-GNP (Figure 3) revealed a blue shift compared to GNP and a red shift compared to intermediate cysteamine-functionalized gold nanoparticles (CysA-GNP). While these changes demonstrate that the functionalization reaction with BA occurred, the spectra also indicate that, in addition to the surface change, an increase in particle size and dispersity may have occurred in the BA-GNP.

### 2.3. FTIR Characterization of GNP and BA-GNP

The BA functionalization of GNP was validated by means of FTIR spectroscopy (Figure 3). The FTIR spectra of GNP are in accordance with previously recorded FTIR data on citrate GNP [20,21] and exhibit the two characteristic symmetric and anti-symmetric stretch vibration peaks of citrate carboxylate at 1593 and 1402 cm^−1^ (also found in the spectra of citrate at 1591, 1419 cm^−1^) as well as the O-H stretch vibration at 3439 cm^−1^ (present in the spectra of sodium citrate at 3452 cm^−1^). The BA-GNP spectra evidently show that BA functionalization of GNP occurred. The majority of BA peaks were found in the spectra of BA-GNP. Among these signals, the most evident are the two C-H stretch vibration peaks at 2943 and 2870 cm^−1^ and the C=O stretch at 1687 cm^−1^ [22]. While both BA and citrate contain OH groups, the O-H stretch vibration for BA-GNP is recorded at 3439 cm^−1^, as in the case of GNP, rather than 3447 cm^−1^ as found in the FTIR spectra of BA, probably indicating that there is residual citrate on the surface of BA-GNP.

### 2.4. Transmission Electron Microscopy (TEM), Dynamic Light Scattering (DLS), Zeta Potential (ζ), Drug Loading Efficiency (DLE) and Gold Content of GNP

**Figure 4:**
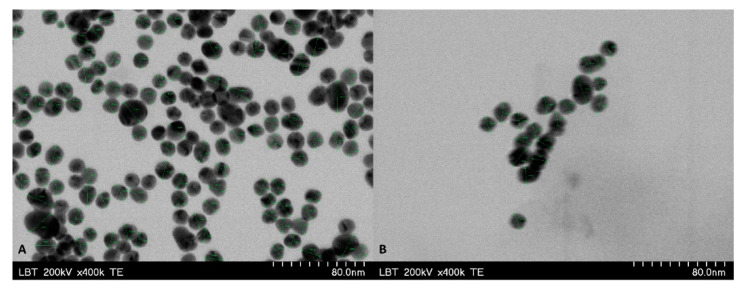
TEM images of GNP (**A**) and BA-GNP (**B**).

TEM analysis revealed stable spherical GNP particles with diameters ranging from 14 to 20 nm (Figure 4). BA-GNP TEM analysis revealed that BA functionalization of GNP had no effect on the shape or diameter of the BA-GNP. However, DLS experiments conducted in aqueous media at neutral pH revealed that BA-GNP increased in hydrodynamic size and had a slightly higher polydispersity index (PDI). The calculated DLE% for BA was approximately 31%, while the recorded ξ potential for both types of assessed nanoparticles was roughly similar (Table 1). The gold content for both samples was determined using X-ray fluorescence (XRF) analysis. Results showed that citrate GNP contains 274,315 ppm of elemental Au, which corresponded to roughly 27.4%, while the Au content for BA-GNP was approximately 14.6%. Both samples also showed traces (<1%) of Na, Si and other metals (Table 2).

**Figure 5:**
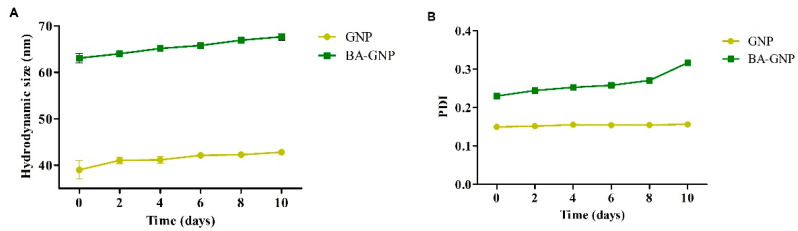
Time course changes of GNP and BA-GNP in (**A**) hydrodynamic size and (**B**) polydispersity index (PDI) stored at room temperature for a 10-day period.

The stability of both citrate and BA conjugated GNP, in aqueous media (pH = 7) was assessed over a 10-day period during which the samples were kept at room temperature, in the dark. Every 2 days, we conducted PDI and ξ potential measurements to record eventual changes in nanosuspension stability. The results are depicted in Figure 5.

### 2.5. Effect of GNP and BA-GNP on Cell Viability and Morphology

**Figure 6:**
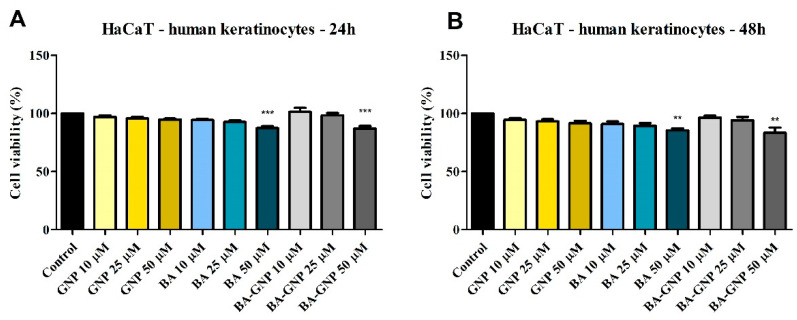
Cell viability of HaCaT cells after 24 h (**A**) and 48 h (**B**) treatment with GNP (diluted to match the same gold content as for BA loaded NPs at 10, 25 and 50 μΜ), BA and BA-GNP (10, 25 and 50 μΜ). The results are expressed as cell viability percentage (%) normalized to control (100%). The data represent the mean values ± SD of three independent experiments performed in triplicate. The statistical differences vs. control was determined using one-way ANOVA analysis followed by Tukey’s multiple comparisons post-test (** *p* < 0.005 and *** *p* < 0.0001).

The compounds were tested for 24 h on HaCaT normal cells using the Alamar Blue assay to determine whether GNP and BA-GNP reduced healthy cell viability (Figure 6). Only the highest concentrations of BA alone and BA-GNP showed low toxicity in normal cells (87.3% and 86.9% HaCaT viable cells, respectively). The results at 48 h did not significantly differ from those obtained at 24 h.

**Figure 7:**
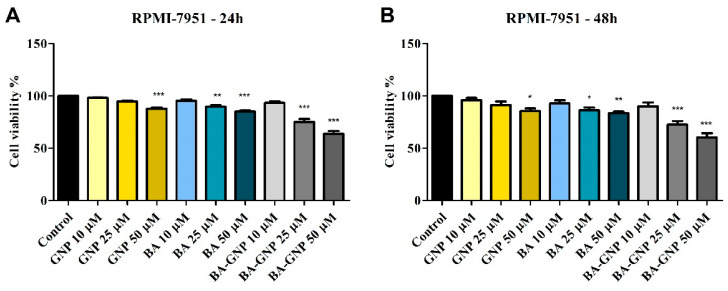
Cell viability of RPMI-7951 cells after 24 h (**A**) and 48 h (**B**) treatment with GNP (diluted to match the same gold content as for BA loaded NPs at 10, 25 and 50 μΜ), BA and BA-GNP (10, 25 and 50 μΜ). The results are expressed as cell viability percentage (%) normalized to control (100%). The data represent the mean values ± SD of three independent experiments performed in triplicate. The statistical differences vs. control was determined using one-way ANOVA analysis followed by Tukey’s multiple comparisons post-test (* *p* < 0.05, ** *p* < 0.005 and *** *p* < 0.0001).

To assess the effect on cell viability, RPMI-7951 human melanoma cells were treated for 24 h with three different concentrations of GNP, BA and BA-GNP (10, 25 and 50 μM) (Figure 7). The results showed that BA-GNP significantly reduced melanoma cell viability at 25 μM (75.1% viable cells) and 50 (63.4%) vs. control (considered to be 100%). Similarly, when tested at 25 and 50 μM, BA reduced the cell viability of RPMI-7951 cells to 89.6% and 85%, respectively. However, only at the highest concentration did GNP significantly inhibit the cell viability (87.6% vs. control) of RPMI-7951. At 48 h, the obtained results also showed no significant differences. As a result, the rest of the experiments were also executed at 24 h.

**Figure 8:**
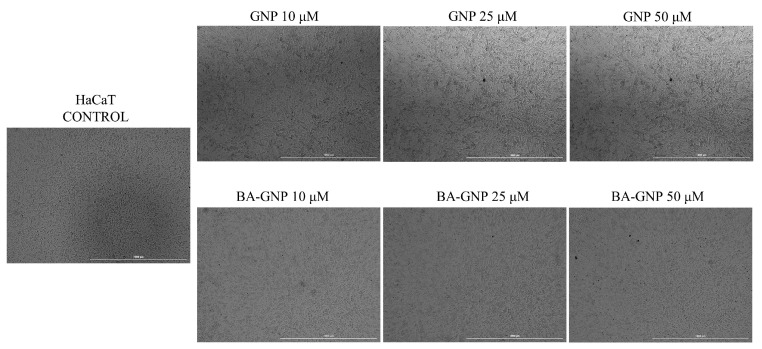
Representative images of the morphological aspect of HaCaT cells after treatment for 24 h with GNP (diluted to match the same gold content as for BA-loaded NPs at 10, 25 and 50 μΜ) and BA-GNP (10, 25 and 50 μΜ). The scale bar was 100 μm.

The effect of GNP and BA-GNP on HaCaT and RPMI-7951 cell morphology changes was studied using an inverted light microscope. Upon analysing the morphological changes induced by the tested compounds on HaCaT cells, no significant changes were observed between control vs. GNP and BA-GNP (10, 25 and 50 μM) treated cells (Figure 8).

**Figure 9:**
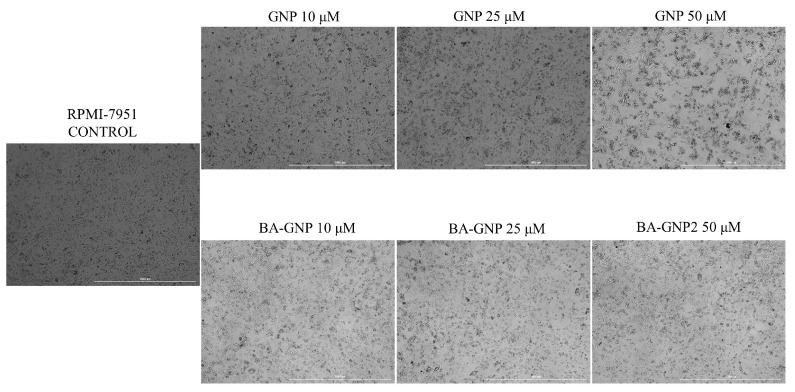
Representative images of the morphological aspect of RPMI-7951 cells after treatment for 24 h with GNP (diluted to match the same gold content as for BA-loaded NPs at 10, 25 and 50 μΜ) and BA-GNP (10, 25 and 50 μΜ). The scale bar was 100 μm.

After 24 h stimulation with GNP (50 μM) and BA-GNP (10, 25 and 50 μM) the results showed that GNP at low concentrations (10 and 25 μM) had no detectable effects on the morphology of RPMI-7951 cells (Figure 4). However, a significant modification in cell morphology (shrinking and shape changes), as well as cell debris and detached cells, was detected at higher concentrations of GNP (50 μM) and for all tested concentrations of BA-GNP (Figure 9); these results suggest their cytotoxic effect against melanoma cells.

### 2.6. Morphological Assessment of Apoptotic Cells by DAPI Staining 

**Figure 10:**
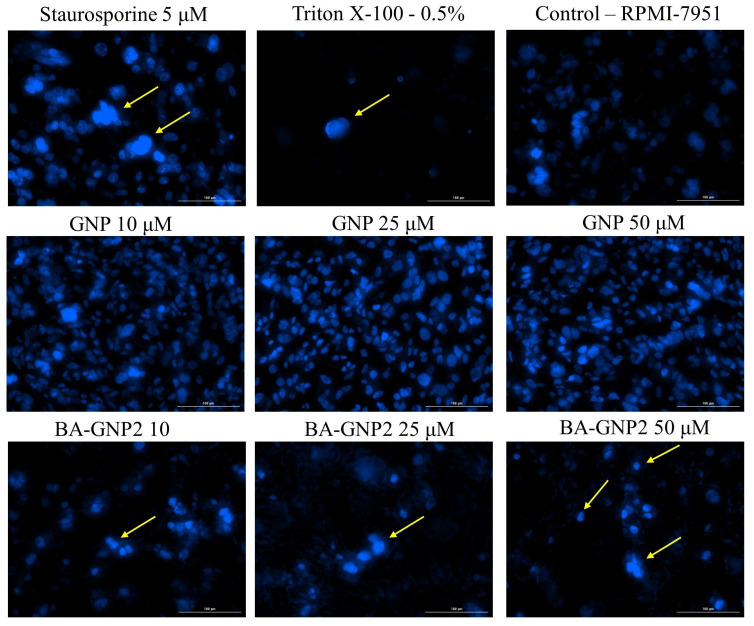
Nuclear staining using DAPI in RPMI-7951 cells after treatment with GNP (diluted to match the same gold content as for BA-loaded NPs at 10, 25 and 50 μΜ) and BA-GNP treatment (10; 25 and 50 μΜ) for 24 h. The pictures were captured 24 h post-treatment. Staurosporine solution (5 μM) and Triton X-100 solution (0.5%) were used as positive controls. The yellow arrows represent signs of apoptosis, such as nuclear shrinkage or nuclear fragmentation. The scale bar is 100 μM.

The cytotoxic activity of a compound can also be highlighted by morphological changes in the nucleus that may indicate the presence of apoptotic and/or necrotic cells. RPMI-7951 nuclei were stained using DAPI dye following a 24 h stimulation with GNP and BA-GNP (10, 25 and 50 μΜ). Apoptotic changes, such as nuclear condensation, shrinkage and fragmentation (yellow arrows), were significantly observed in RPMI-7951 cells treated with all concentrations of BA-GNP, the most visible effect being recorded at a concentration of 50 μM (Figure 10).

From the presented results, it is clear that BA-GNP in high concentrations (25 and 50 μΜ) exerts a cytotoxic effect on RPMI-7951 melanoma cells by significantly decreasing cell viability and inducing morphological apoptotic features.

### 2.7. Irritation Potential of BA-GNP Using the HET-CAM Assay

**Figure 11:**
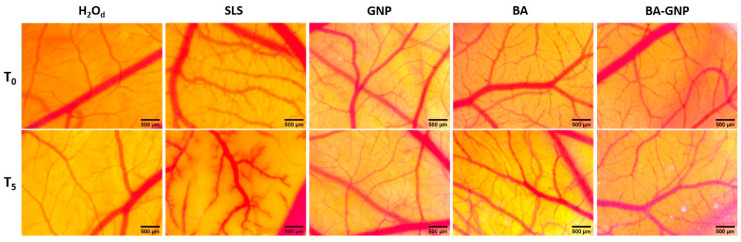
Irriitation potential assessment using the HET-CAM method. Stereomicroscope images show the in-face chorioallantoic membrane before (T_0_) and 300 s after application (T_5_) of 300 μL of the test sample in concentrations of 1 μM and control samples (distilled water H_2_O as negative control, SLS 0.5% as positive control and DMSO 0.5% as solvent control); scale bars represent 500 μm.

Using the HET-CAM in vivo assay, we investigated the potential irritability of BA-GNP on mucosal tissues. Thus, the impact on three parameters of the vascular plexus, namely hemorrhage, lysis and coagulability, was assessed on the developing chorioallantoic membrane of the chicken embryo. Distilled water served as a non-irritant control, while SLS 0.5% served as a strong irritant, with an IF of 15.77 on the Luepke scale: 0–0.9—non-irritant, 1–4.9 weak irritant, 5–8.9 moderate irritant, 9–21 strong irritant. All tested samples were found to be non-irritant, with no adverse effects on any of the three vascular parameters (Table 3, Figure 11). As a result, GNP can be considered biocompatible with mucosal tissues and a safe type of BA nanoformulation for local applications.

### 2.8. GNP and BA-GNP Effects on HaCaT and RPMI-7951 Mitochondrial Respiration

**Figure 12:**
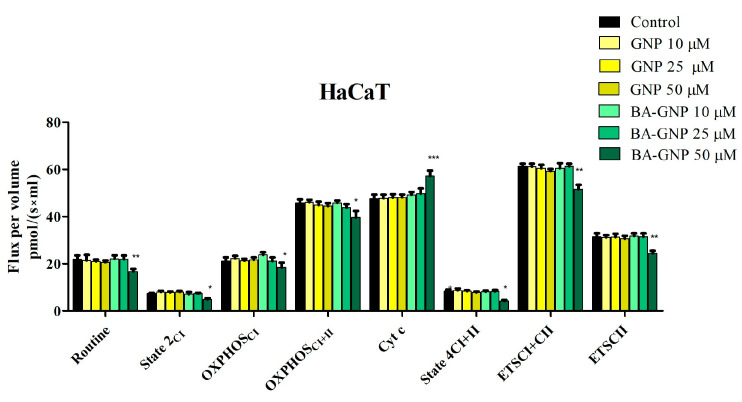
Mitochondrial respiration of immortalized human keratinocytes—HaCaT cells after 24 h treatment with GNP (diluted to match the same gold content as for BA-loaded NPs at 10, 25 and 50 μΜ)) and BA-GNP (10, 25 and 50 μΜ). Data represent the mean ± SD of five individual experiments. Values with *p* < 0.05 were considered to have a statistically significant difference (* *p* < 0.05, ** *p* < 0.01 and *** *p* < 0.01). The respiratory parameters displayed represent the following: Routine—respiration of cells suspended in a substrate-free media; State 2_CI_: CI-driven mitochondrial respiration in basal conditions—LEAK, OXPHOS_CI_—active respiration dependent on CI substrates and exogenous ADP; OXPHOS_CI+II_ –maximal active respiration driven by both CI and CII; Cyt c—evaluation of mitochondrial membrane integrity; State 4_CI+II_—LEAK respiration dependent on both CI and CII; ETS_CI+II_—maximal respiratory capacity of the electron transport system in the fully noncoupled state; ETS_CII_—electron transport system maximal capacity dependent only on CII.

Numerous studies have identified mitochondria as a target in BA and GNP anticancer mechanisms of action [23,24] and also mitochondria’s involvement in activating apoptosis in mammalian cells [25]. Thus, to comprehend the expanded effect on mitochondrial function given by the association of GNP with BA, three concentrations of GNP and BA-GNP (10, 20 and 50 μM) were tested on permeabilized HaCaT and RPMI-7951 cells using high-resolution respirometry. The values recorded showed that, in HaCaT cells, the 24 h treatment with the highest concentration (50 μM) of BA-GNP was able to significantly inhibit all mitochondrial respiratory rates (basal respiration—routine/State2/State4, active respiration—OXPHOS and maximal respiration in the uncoupled state—ETS) driven solely by CI but also by both CI+CII when working conjunctively (Figure 12). In parallel, a significant increase in the Cyt c rate was observed, suggesting that BA-GNP impairs the outer mitochondrial membrane integrity of HaCaT cells at 50 μM (Figure 12).

**Figure 13:**
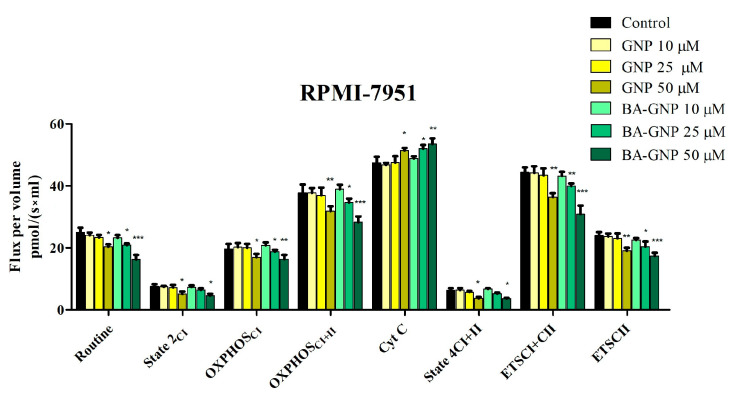
Mitochondrial respiration of skin malignant melanoma cells—RPMI-7951 cells after 24 h treatment with GNP (diluted to match the same gold content as for BA loaded NPs at 10, 25 and 50 μΜ) and BA-GNP (10, 25 and 50 μΜ). Data represent the mean ± SD of five individual experiments. Values with *p* < 0.05 were considered to have a statistically significant difference (* *p* < 0.05, ** *p* < 0.01 and *** *p* < 0.01). The respiratory parameters displayed represent the following: Routine—respiration of cells suspended in a substrate-free media; State 2_CI_: CI driven mitochondrial respiration in basal conditions—LEAK, OXPHOSCI—active respiration dependent on CI substrates and exogenous ADP; OXPHOS_CI+II_—maximal active respiration driven by both CI and CII; Cyt c—evaluation of mitochondrial membrane integrity; State 4_CI+II_—LEAK respiration dependent on both CI and CII; ETS_CI+II_—maximal respiratory capacity of the electron transport system in the fully noncoupled state; ETS_CII_—electron transport system maximal capacity dependent only on CII.

In RPMI-7951 melanoma cells, the data showed that BA-GNP produced a significant reduction of all respiratory rates together with an increase in the Cyt c rate (Figure 13). Moreover, BA-GNP inhibits active respiration (OXPHOS_CI_ and OXPHOS_CI+CII_) and the maximal respiratory capacity of the electron transport chain (ETS_CI_ and ETS_CI+CII_), when tested at both 25 and 50 μΜ, thus causing impairment of mitochondrial function. The compound produced a similar decrease in routine respiration when tested at 25 and 50 μΜ on RPMI-7951 melanoma cells. Despite an observed dose-dependent decrease, only at 50 μΜ BA-GNP was able to inhibit LEAK respiration (State 2_CI_ and State 4_CI+II_) in a statistically significant manner, suggesting that the compound was able to decrease the proton leak/slip and thus did not produce a mitochondrial uncoupling effect. GNP elicited a similar effect on RPMI-7951 cells but only at 50 μΜ, the highest tested concentration (Figure 13).

### 2.9. Western Blot

**Figure 14:**
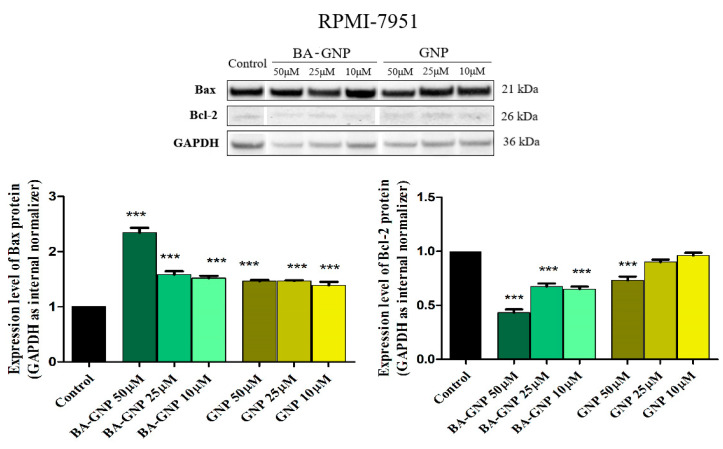
Determination of Bax and Bcl-2 protein expression in RPMI-7951 cells. Protein extracts from whole cells were prepared and equal amounts of protein (25 µg per lane) were separated by Bolt™ 4–12%, Bis-Tris, and polyacrylamide gels. After transfer on the nitrocellulose membrane, immunodetection was carried out using anti-Bcl-2 and anti-Bax antibodies. The data represent the mean values ± SD of three independent experiments after normalization against the GAPDH loading control and the control group. The statistical differences vs. control were determined using one-way ANOVA analysis followed by Tukey’s multiple comparisons post-test (*** *p* < 0.0001).

Mitochondria-dependent apoptosis is regulated by both pro-apoptotic Bcl-2 associated X (Bax) and anti-apoptotic B-cell lymphoma 2 (Bcl-2), two well-studied members of the large Bcl-2 family. Thus, to gain insights regarding the exact mechanism responsible for GNP and BA-GNP cytotoxic effects and, more precisely, to delineate the mitochondrial apoptotic pathway from the apoptotic pathway induced by DNA damage, Bax and Bcl-2 protein expression levels were determined using Western blot (Figure 13). The expression of Bax was upregulated upon treatment with both GNP and BA-GNP (Figure 13). Graphical analysis of Bax expression levels after loading control normalization showed that all concentrations of GNP and BA-GNP were able to significantly increase the protein expression vs. control, with the highest increase being observed upon RPMI-7951 treatment with BA-GNP in the highest concentration (50 μM). An inversely correlated dose-dependent response was observed in the case of anti-apoptotic Bcl-2 expression level. Treatment with increasing concentrations of BA-GNP led to a more drastic inhibition of Bcl-2 expression level; the strongest inhibition was once more observed for BA-GNP-treated cells (Figure 14). GNP alone was able to produce a similar effect on Bcl-2 protein expression only at 50 μM (Figure 14).

## 3. Discussion

Melanoma is the most aggressive type of skin cancer, accounting for approximately for 1 in 5 skin cancers; according to the International Agency for Research on Cancer (IARC), the annual number of melanoma new cases will increase by more than 50% until 2040 [26]. Despite numerous signs of progress achieved in clinical settings, including targeted therapies, melanoma treatment still has many challenges due to the occurrence of drug resistance and the severity of side effects, thus requiring new alternatives. Betulinic acid was extensively investigated as an anticancer agent, starting with the first report in 1976, when Trumbull et al. identified the compound as an anti-lymphocytic leukemia agent [6]; more importantly, BA acts as a selective inhibitor of human melanoma, as reported by Pisha et al., thus avoiding the cytotoxic alteration of healthy cells [4]. The current study approached a common drawback in the investigation of pentacyclic triterpenes, the poor BA bioavailability, which may be solved by its conjugation to GNP. GNP can be synthesized easily in various shapes and sizes, which determine their optical and electrical properties, are biocompatible and, additionally, their negative surface charge allows their functionalization by drugs and targeting ligands [27].

In the current work, GNP were synthesized by the well-established citrate reduction method of chloroaurate, which afforded stable spherical-shaped GNP with a diameter range roughly between 14 and 20 nm. This result is consistent with the employed method, as it was previously reported by numerous studies [28,29,30]. BA-GNP were obtained by coupling BA onto the surface of cysteamine functionalized GNP, using a water-soluble carbodiimide (EDC) for the coupling reaction. BA-GNP formation was observable due to the blue shift recorded in both the physical aspect of the nanosuspension (color change) and the UV-VIS spectra of the obtained nano-formulation. However, the clearest indicator of BA-GNP formation was revealed through FTIR analysis, which showed that BA-specific vibration peaks also appeared in the BA-GNP spectra. BA functionalization did not affect the TEM-recorded size of the nanoparticles, but an increase in the hydrodynamic diameter was observed. BA functionalization also induced a slight increase in the dispersity of the obtained nanoparticles. The ζ potentials were recorded in water at a neutral pH. Similar values were obtained for both samples (−29.4 mV for GNP and −27.2 for BA-GNP). The recorded ζ potentials for our GNP conjugates are consistent with stable GNP that have a low probability of forming aggregates over time, similar to previously reported findings [31]. Citrate GNP showed a gold content of 27.4%, while BA-GNP gold content was lower (17.4%). This reduction in gold content, relative to the total sample mass, can be attributed to the replacement of citrate residues with BA, which has a higher molecular weight compared to citrate. Taking into account that XRF determination searched for elements from Na to U, the difference in percentage that was not accounted for can be attributed to the organic coating of the GNP surface, which would be 71% for citrate GNP and 84.4% for BA-GNP. Similar gold content values for citrate GNP were reported by Borse et al., where synthesized citrate GNP contained approximately 29% gold [32]. Additionally, regarding the organic coating, the drug-loading efficiency was determined by quantifying the BA content from the obtained BA-GNP. Our results showed a DLE of 31%. These results are in line with the findings of Oladimeji et al., which reported the synthesis of BA-functionalized epigallocatechin gallate (EGCG)—capped GNP [17]. In their work, the authors stated that BA was grafted onto the surface of GNP by means of Steglich esterification of the EGCG’s OH groups with BA, using EDC. As in our case, BA’s presence on the surface of GNP was clearly observed through FTIR analysis. The shapes, sizes, physical behavior, and DLE of their obtained BA-loaded EGCG-GNP were highly similar to our results. The work reported a 15-nm average-sized spherical GNP upon which BA functionalization had no effect regarding average size, with an observed blue shift in the UV-VIS spectra compared to EGCG-GNP and a DLE% of 25.4%. Our obtained hydrodynamic size was much lower than the results reported by Oladimeji et al., but this is probably caused by the fact that their particles have very voluminous compounds linked to the surface, in the form of large PEG residues, apart from EGCG-BA esters [17]. Sadly enough, to the best of our knowledge, this is the only other piece of literature that physico-chemically describes a triterpenic acid-functionalized GNP formulation. Citrate and BA conjugated GNP proved to be stable over a 10-day period where a slight increase in PDI and hydrodynamic size was observed for both samples. However, the recorded changes pertaining to the above-mentioned parameters showed a higher increased frequency for BA-GNP, meaning that this nanoformulation is somewhat less stable than citrate-capped GNP. Additionlly, during this 10-day period, no visible changes regarding the physical aspects of both samples occurred. This was expected since citrate-conjugated GNP are known to be stable for longer periods of time than our 10-day observed time interval [32]. With regard to BA-GNP, no available literature regarding the stability of any triterpene-functionalized GNP is available. 

As previously mentioned, the main features of GNP are firmly associated with their size and shape; accordingly, small particles (<100 nm) are able to penetrate the skin’s deep layers and also achieve higher bioavailability compared to bigger sizes. In fact, for spherical GNP with diameters within the nanomolar range, experiments revealed a maximum cellular uptake for 50 nm NPs, a size that mimics viruses and lipid-carrying proteins [33].

The cytotoxic effects of the BA-GNP were assessed against BA alone in human melanoma RPMI-7951 cells, which can be described as epithelial-like adherent cells able to synthesize and transport melanin and harbor BRAF, PTEN and TP53 mutant genes [34]. The Alamar Blue oxidation-reduction indicator, which quantifies cell viability by changing color in response to chemical reduction due to cell growth, revealed a significant dose-dependent cell death percentage after the application of BA-GNP samples; by contrast, only the highest GNP concentration was able to slightly inhibit cell growth. Of note, cell viability decreased more pronouncedly when conjugated BA was tested compared to the BA alone, thus indicating that the presence of the phytocompound indeed plays a key role in the inhibitory process and that its penetration into the cancer cells is facilitated by the metallic nanocarrier. In addition, GNP might still contribute to the overall cytotoxic effect since Lu et al. reported the dose-dependent anti-melanoma activity of such nanoparticles in B16F10 mouse melanoma cells exerted both through cytotoxic effects as well as by reducing the motility and migration of melanoma cells [35]. The cytotoxic activity of phytocompound-conjugated GNP against melanoma cells was also reported for betulin [7] while other phytocompounds such as hesperidin [36] were loaded on GNP as anticancer agents against various cell lines. A second cell viability experiment was conducted in human keratinocytes (HaCaT) in order to highlight a potential selective anti-melanoma activity; indeed, experimental data not only revealed the lack of cytotoxic effects after the application of low concentrations of BA-GNP but even the occurrence of a slight stimulatory activity. However, when the tested sample contained the highest BA-GNP concentration, it induced a mild decrease in cell viability, which is in agreement with the selective intrinsic behavior of BA manifested in various cancer cell lines [6]. Similarly, naked GNP did not exert any cytotoxic effects on normal keratinocytes except for the highest tested concentration, but even in this case, the overall effect was insignificant. The reported results are in agreement with previously published data on GNP, which showed cytotoxic activity in HaCaT cells only when the smallest sizes (1–3 nm) were used, bigger sizes being basically non-toxic [35].

BA recently revealed a dose-dependent inhibitory effect on mitochondrial respiration and glycolysis in human melanoma cells when applied in sub-toxic concentrations [24]. Moreover, its cytotoxic effects are characterized by apoptotic features, as indicated by morphological cell alterations and the upregulation of pro-apoptotic markers. In the current study, BA-GNP was applied to normal and cancer cells, leading to distinct results in terms of cell morphology. In melanoma cells, all concentrations of the phytocompound-conjugated GNP induced specific alterations of cell morphology, such as shrunk and detached cells, as well as cell debris, while only the highest concentration of naked GNP caused such alterations. By contrast, in normal human keratinocytes, no cellular morphological changes were recorded for either bare or conjugated GNP; in addition, betulinic acid alone did not reduce HaCaT cell viability, thus validating the literature data. These findings confirmed the occurrence of cytotoxic effects of BA-GNP against the tested melanoma cells in a selective manner, which enables their employment as antitumor agents. However, for bare GNP, the reported results are in contradiction with data published by Schaeublin et al., who revealed that neutral GNP may cause a certain level of cytotoxicity in HaCaT cells through necrosis induction [37]. Taking a closer look at literature data, one can notice controversial results in terms of GNP cytotoxicity against normal skin cells, results that can be attributed to the inherent differences in the study design in terms of dosages, surface chemistry, cell types, etc. [38].

Considering the previously reported pro-apoptotic activity of BA, the current study attempted to assess if the same underlying mechanism is preserved for the BA-GNP; thus, melanoma cells were submitted to DAPI staining in order to identify the presence of apoptotic and/or necrotic cells. The DAPI (4′,6-diamidino-2-phenylindole) dye has the ability to strongly bind to the adenine-thymine rich regions in DNA while emitting blue fluorescence in direct relation to the DNA amount; the DAPI staining allows the identification of apoptotic cells that exhibit chromosomal condensation and fragmentation as well as nuclear blebbing [39]. Following 24 h stimulation with either bare or BA-GNP, RPMI-7951 nuclei were stained with DAPI dye; apoptotic changes such as nuclear condensation, shrinkage and fragmentation in a dose-dependent manner were revealed only for the phytocompound-conjugated GNP, while the naked GNP left the nuclei unaltered. All changes were validated using staurosporine as a positive control. Apoptosis is a highly regulated programmed cell death with a critical role in homeostasis and whose deregulation leads to a wide range of diseases; delayed or inhibited apoptosis occurs in cancer and autoimmune pathologies and is also responsible for the development of drug resistance [40]. Betulinic acid is well-known as an apoptotic inducer via the mitochondrial intrinsic pathway; although spherical GNP were reported to induce apoptosis in osteosarcoma cells [41] and gold nanostars had similar effects in melanoma cells [42], in the current study the bare spherical GNP lacked any sign of pro-apoptotic effect. Therefore, one may conclude that the biological effects of GNP depend on their size as well as on the specific tested cell line; for the BA-GNP synthesized within the current experiments, the apoptotic cellular effects are triggered by the phytocompound, while the metallic nanocarrier presumably facilitates cell penetration.

The intrinsic or mitochondrial apoptotic pathway can be triggered by pentacyclic triterpenes, including betulinic acid, which thus acts as mitocan (mitochondria targeting anticancer agent) [43]. High-resolution respirometry studies were conducted in both melanoma cells and human keratinocytes in order to assess the underlying mechanism of action for the BA-GNP; we used the same protocol that we previously applied for the BA alone, which indicated that triterpenic acid is able to inhibit cellular respiration by inhibiting both basal and active respiration as well as maximal uncoupled respiration [24]. One important difference between malignant and normal cells is the pathway to produce ATP molecules in order to supply the necessary energy for metabolic processes; while cancer cells mainly rely on glycolysis, even when enough oxygen is available, healthy cells use oxidative phosphorylation, i.e., the OXPHOS process [44]. However, tumors appear to contain small populations of quiescent cells greatly depending on mitochondrial respiration, which exhibit proliferative potential and may be responsible for tumor drug resistance [45]; these cells may become the target of mitocans, such as BA and its pharmaceutical formulations. Moreover, recent studies revealed that certain types of naturally resistant melanomas to targeted therapies appear to depend on mitochondrial respiration rather than glycolysis [46]; in order to sustain their proliferation, invasion and distal migration, melanoma cells are able to regulate multiple metabolic pathways, including glycolysis and OXPHOS [47]. Subsequently, targeting the OXPHOS metabolic pathway may render melanoma cells highly sensitive to cytotoxic compounds, thus providing future strategies for cancer treatment. Experimental data showed that neither BA-GNP nor naked GNP had any significant influence on mitochondrial respiration in HaCaT cells when used in low concentrations; however, the highest concentration (50 μM) of BA-GNP applied for 24 h significantly inhibited all mitochondrial respiratory rates driven either by CI alone or by both CI+CII conjunctively. The parallel increase in the Cyt c rate indicates that this concentration of BA-GNP is able to alter the outer mitochondrial membrane integrity of HaCaT cells.

Mitochondrial uncoupling, which diminishes the physiological link between mitochondrial respiration and ATP synthesis, favors the metabolic adaptation of cancer cells [48]; Coricovac et al. reported that BA does not act as a classical uncoupler since it induces mitochondrial dysfunction without being able to mediate proton leak [24] which would be noticed as increased State 2_CI_ and State 4_CI+CII_ respiratory rates.

In RPMI-7951 melanoma cells, data showed that BA-GNP produced a significant reduction of all respiratory rates together with an increase of Cyt c rate; as the routine state represents the physiological control of cellular substrate uptake, intermediary metabolism and energy turnover, its decrease produced by BA-GNP suggests that this compound can decrease mitochondrial ATP demand and alter mitochondrial function. Further, the decrease in State 2_CI_ and State 4_CI+CII_ shows a decrease in oxygen consumption when the phosphorylation system is inactive by decreasing the proton leak/slip across the inner mitochondrial membrane. Moreover, BA-GNP inhibits active respiration (OXPHOS_CI_ and OXPHOS_CI+CII_) and also the maximal respiratory capacity of the electron transport chain (ETS_CI_ and ETS_CI+CII_), thus demonstrating impairment of mitochondrial function. Similar effects were elicited by naked GNP in RPMI-7951 cells, but only when the highest concentration was used. The mitochondrial-targeted activity of BA is supported by many studies that have demonstrated its ability to induce apoptosis via a direct effect on mitochondria [43,49,50]. GNP ability to directly target and enter mitochondria is severely limited by size and type; however, GNP translocation across the mitochondrial membranes is not required in order to alter mitochondrial function, as enhanced ROS production, mitochondrial membrane potential and morphological changes were reported after GNP treatment [51]. Another aspect is the negative feedback connection between proton leak (uncoupling) and ROS generation; in the current study, the decrease in State 2_CI_ and State 4_CI+CII_ may indicate that BA-GNP can trigger mitochondrial ROS production, which in turn may induce cancer cell apoptosis [52]. Given the fact that BA was previously reported to induce ROS-mediated apoptosis in certain cancer cells [53] one may presume that ROS generation is indeed triggered by the BA-conjugated GNP.

Pentacyclic triterpenes were shown to induce apoptosis via the intrinsic pathway, which evolves through the balanced activation of pro-(Bax, Bak) and anti-apoptotic (Bcl-2, Bcl-X, etc.) proteins; specifically, there are numerous studies [54] including our own [24] that identify betulinic acid as an apoptosis inducer through the upregulation of the pro-apoptotic Bax and the downregulation of the anti-apoptotic Bcl-2 proteins. Following conjugation with GNP, the resulting nanoformulation acted in a similar manner. Although pro- and anti-apoptotic protein expression was also significantly influenced by GNP alone at the highest concentration used, these results are only comparable with the lowest concentration of BA-GNP, suggesting that GNP conjugation has an apparent synergistic effect toward Bcl-2 and Bax expression, but the main pro-apoptotic effect is mainly due to BA. These results are in line with the previously reported effects of BA alone, thus suggesting its potential use as a mitocan against RPMI-7951 melanoma cells.

## 4. Materials and Methods

### 4.1. Synthesis of Citrate-Capped Gold Nanoparticles (GNP)

Citrate-capped gold nanoparticles were obtained by the reduction of chloroauric, HauCl_4_, (Merck KGaA, Darmstadt, Germany) acid using trisodium citrate dihydrate (Merck KGaA, Darmstadt, Germany) [7]. Specifically, HAuCl_4_ (0.2 mmoles, 68 mg) was dissolved in 180 mL of deionized water with constant stirring. The solution was brought to a boil, and a 20 mL solution of sodium citrate dihydrate (0.6 mmoles, 176.5 mg) was added. The heat was turned off when the solution’s color turned ruby red, after which it was allowed to cool to room temperature for 24 h. GNP formation was confirmed through UV-VIS spectroscopy. GNPs were purified using successive centrifugations at 13,000× *g*, water removal, and resuspension in water (4 cycles) until the pH of the obtained nanosuspension was neutral to ensure the removal of excess unreacted citrate.

### 4.2. Synthesis of BA-Loaded Gold Nanoparticles (BA-GNP)

BA-functionalized gold nanoparticles were obtained using cysteamine (Merck KGaA, Darmstadt, Germany) as a cross-linker between BA’s COOH (linked through an amidic bond to cysteamine’s NH_2_ group) and the surface of GNPs (linked through a thiolate-gold bond to cysteamine’s SH group). A 10 mL nanosuspension of previously synthesized GNP was loaded with 0.02 mmoles (1.54 mg) cysteamine under continuous stirring. The suspension instantly became blue, suggesting that thiolate-gold bond formation and implicit cysteamine binding to the GNP surface occurred. The now cysteamine-functionalized GNP suspension was loaded with 0.03 mmoles (5.75 mg) of N-(3-Dimethylaminopropyl)-N′-ethylcarbodiimide hydrochloride (EDC. Merck KGaA, Darmstadt, Germany) and was stirred until EDC was completely dissolved, after which 0.02 mmoles BA (9.13 mg) were finally added. The suspension was sonicated for 1 h (0.8 cycles, 80% amplitude) using a UP200S ultrasonic homogenizer (Hielscher Ultrasonics GmbH, Teltow, Germany) equipped with an adequate volume sonotrode. The sonication step was considered complete when no observable traces of BA were left floating on the surface of the GNP nanosuspension. After this sonication cycle, the color of the GNP nanosuspension reverted from blue to purple and was stirred at room temperature for 24 h. The obtained BA-GNPs were extracted with ethyl acetate (4 × 5 mL) (Merck KgaA, Darmstadt, Germany) in order to remove unreacted BA. The pH of the obtained nanosuspension was slightly acidic, probably due to unreacted EDC chloride. The suspension was then submitted to the purification process described for citrate-capped GNPs (Section 4.1), which also removed the water-soluble acyl-urea byproduct and afforded a final nanosuspension with a neutral pH.

### 4.3. Characterization of GNP and BA-GNP (UV-VIS, FTIR, TEM, Hydrodynamic Size, Zeta Potential)

The formation of GNP and BA-GNP was monitored using UV-VIS spectrometry. The adsorption spectra of the samples were measured using a Shimadzu UV-1900i spectrophotometer with a wavelength range of 400–800 nm. Fourier Transform Infrared spectrometry (FTIR) was used to determine the bond formation and functionalization of the triterpene-loaded GNP. To produce dried GNP samples, aliquots of 3 mL GNP and BA-GNP were centrifuged (13,000× *g*) and oven-dried at 80 °C for 24 h. All spectra were recorded using potassium bromide pellets on a Shimadzu IR Affinity-1S spectrophotometer in the 400–4000 cm^−1^ range (4 cm^−1^ resolution). Transmission electron microscopy (TEM) was used to assess particle size and was conducted using a Hitachi HD2700 cold field emission gun STEM (Chiyoda, Tokyo, Japan) outfitted with two windowless EDX detectors (X-MaxN 100). The Vasco Particle Size Analyzer and the Wallis Zeta-potential Analyzer (Cordouan Technologies, Pessac, France) were used to measure the size and stability of the aqueous solutions in the samples that were studied. Samples were analyzed at pH = 7 in aqueous media. For data acquisition, the following size analyzer parameters were preset: DTC position down, temperature 25 °C, 90% laser power, number of channels 339, time interval 16 milliseconds, acquisition mode continuous, analysis mode cumulants, and for the potential analyzer: 25 °C, 80% laser power plastic cuvette, 5 electrode distance, medium resolution, Henry function Huckel.

### 4.4. Drug Loading Efficiency Determination

The drug-loading efficiency of BA-GNP nanoformulations was achieved by means of LC-MS spectroscopy, using a 6120 LC-MS analytical system (Agilent, Santa Clara, CA, USA) consisting of a 1260 Infinity HPLC equipped with G1322A degasser, G1311B quaternary pump, G1316A column thermostat, G1365C MWD detector, and G1328C manual injector coupled with a Quadrupolar (Q) mass spectrometer equipped with electrospray ionization source (ESI). A 10 mL sample of BA-GNP was subjected to treatment with NaOH 4 M for 24 h in order to hydrolyze BA from the cysteamine linker. The suspension was brought to a neutral pH using concentrated HCl and was further extracted with ethyl acetate. The solvent was subsequently removed from the organic fractions and the residue was redissolved in methanol. BA concentration was determined using a 7-point plot curve by conducting a previously described method [55]. Drug loading efficiency (*DLE*) was calculated using the following formula:

$$DLE\%=\frac{We}{Wt}\times 100$$

where

*We* = quantity of the encapsulated drug, spectrometrically determined

*Wt* = quantity of total drug added to the nanoformulation. 

### 4.5. Gold Content Determination of GNP Using X-ray Fluorescence (XRF) Analysis

Experiments on GNP samples were carried out using a wavelength dispersive X-ray fluorescence (X-MET8000 series, HHXRF models) spectrometer (Hitachi, Chiyoda, Japan). The calibration of the X-MET8000′s fundamental parameters (FP) was optimized for the analysis of GNP samples. The samples were air dried for 48 h before being ground and sieved with a 0.1 mm mesh sieve to ensure a uniform particle size. Prior to analysis with an X-ray fluorescence (XRF) spectrometer, each sample was labeled and stored in a dry plastic container that had been precleaned with concentrated nitric acid. According to the literature, samples were measured using a portable stand to provide complete user protection against scattered radiation [56]. The results are given in ppm values and weight percentage (wt%).

### 4.6. Cell Culture

The immortalized human keratinocytes—HaCaT and human skin malignant melanoma cells—RPMI-7951 were purchased from CLS Cell Lines Service GmbH (Eppelheim, Germany) and American Type Culture Collection (ATTC, Lomianki, Poland), respectively. The cells were received as frozen items and stored in liquid nitrogen. HaCaT cell lines were cultured in Dulbecco’s modified Eagle Medium (DMEM) high glucose, while RPMI-7951 cells were cultured in Eagle’s Minimum Essential Medium (EMEM). Both mediums were supplied with a mixture of 10% fetal bovine serum (FCS) and 1% a combination of 100 U/mL penicillin and 100 U/mL streptomycin (Sigma-Aldrich, Munich, Germany). The cell lines were maintained in a humidified incubator with 5% CO_2_ at 37 °C and used for treatment with the tested compounds after reaching 85–90% confluency. Cells were treated with GNP (10, 25 and 50 μΜ) and BA-GNP (10, 25 and 50 μΜ) for 24 h. The cell number was determined in the presence of Trypan blue using an automated cell counting device (Thermo Fisher Scientific, Inc., Waltham, MA, USA).

### 4.7. Cell Viability Assessment—Alamar Blue Assay and Cell Morphology

#### 4.7.1. Alamar Blue Staining 

The cytotoxic activity of GNP (10, 25 and 50 μΜ) and BA-GNP (10, 25 and 50 μΜ) on HaCaT and RPMI-7951 cell lines was assessed by means of Alamar Blue assay. The cells (1 × 10^4^ cells/well) were seeded in a 96-well plate and allowed to attach for 24 h. The next day, the culture medium was replaced with fresh medium and the attached cells were stimulated with the tested compounds. After 24 h, 20 μL of Alamar Blue reagent was added to each well and the plates were incubated again for 3 h at 37 °C. Absorbance was measured at 570 and 600 nm using a microplate reader (xMark™ Microplate Spectrophotometer, Biorad, Hercules, CA, USA).

#### 4.7.2. Cellular Morphology Evaluation 

The effect of GNP (10, 25 and 50 μΜ) and BA-GNP (10, 25 and 50 μΜ) on HaCaT and RPMI-7951 morphology was assessed immediately after the treatment (0 h) with the compounds and at 24 h post-treatment. The images were captured and analyzed using the cellSens Dimensions v.1.8. Software (Olympus, Tokyo, Japan).

### 4.8. Immunofluorescence Assay

The morphological assessment of HaCaT and RPMI-7951 apoptotic cells treated with GNP (10, 25 and 50 μΜ) and BA-GNP (10, 25 and 50 μΜ) was performed using the 4, 6′-Diamidino-2-Phenylindole (DAPI) Staining. HaCaT and RPMI-7951 cells were seeded in 6-well plates (5 × 10^6^ cells/ well). After reaching 85–90% confluence, the old medium was replaced with fresh medium and the cells were stimulated with GNP (10, 25 and 50 μΜ) and BA-GNP (10, 25 and 50 μΜ) and incubated for 24 h at 37 °C. After the incubation period, the cells were washed 2–3 times with cold phosphate-buffered saline PBS (1X) (Thermo Fisher Scientific, Boston, MA, USA) and fixed with 4% paraformaldehyde in PBS. In the next step, the cells were permeabilized with Triton X/PBS 2% for 30 min at room temperature, washed 2–3 times with cold PBS and blocked with 30% FCS in 0.01% Triton X for 1 h at room temperature. In the end, the cells were washed again 2–3 times with cold PBS, stained with DAPI (300 nM) and incubated at 4 °C in the dark overnight. Nuclear alterations were analyzed using the integrated DP74 digital camera of an Olympus IX73 inverted microscope (Olympus, Tokyo, Japan).

### 4.9. HET-CAM Assay

The in vivo Hen’s Egg Chorioallantoic Membrane Test (HET-CAM) assay determines a potential irritant effect on the vascular plexus of the chorioallantoic membrane [57]. The HET-CAM method was carried out in accordance with the ICCVAM recommendations published in Appendix G in November 2016 and adapted to our conditions [58,59,60]. Thus, a volume of 300 μL of control or test solution was applied, and the modifications produced at the CAM level were monitored using stereomicroscopy (Discovery 8 Stereomicroscope, Zeiss, Göttingen, Germany), registering significant images (Axio CAM 105 color, Zeiss, Göttingen, Germany) before application and after 5 min of contact with the samples. All images were processed with Axio CAM 105 color, Zeiss, Göttingen, Germany, Gimp 2.8 (GNU Image Manipulation Program Development Team, v 2.10.24, https://www.gimp.org/; accessed on 2 September 2022), and ImageJ software (Image J Version 1.53k, https://imagej.nih.gov/ij/index.html; accessed on 2 September 2022, NIH, Bethesda, MD, USA). The negative control was distilled water, with the solvent control DMSO 0.5%, and the positive control was sodium lauryl sulfate (SLS) 0.5% aqueous solution. GNP and BA-GNP were tested at 1 μM concentrations. The produced reactions were observed for 5 min (300 s), and the time at which each reaction occurred was recorded in seconds. Finally, the following reactions were observed: hemorrhage—H (blood vessel bleeding), vascular lysis—L (disintegration of blood vessels), and coagulation—C. (intra- or extra-vascular protein denaturing). A variety of analysis methods can be used to determine the irritancy potential of test substances. An irritation score (IS) is a frequently employed method of analysis. An *IS* value was generated using the following formula:

$$IS=5\times \frac{301-Sec\;H}{300}+7\times \frac{301-Sec\;L}{300}+9\times \frac{301-Sec\;C}{300}$$

where *H* = hemorrhage; *L* = vessel lysis; *C* = coagulation; hemorrhage time (*Sec H*) = onset of hemorrhage reactions on CAM (in seconds); lysis time (*Sec L*) = onset of vessel lysis on CAM (in seconds); coagulation time (*Sec C*) = onset of coagulation formation on CAM (in seconds); coagulation time (*Sec C*) = onset of (in seconds). Means were calculated. The formula includes a factor indicating the impact of the observed effect on vascular damage; for example, coagulation has the greatest impact, as expressed by multiplication factor 9. *IS* values ranged from 0 to 21 on a scale of 0 to 21.

### 4.10. High-Resolution Respirometry

High respirometry studies (Oxygraph-2k Oroboros Instruments GmbH, Innsbruck, Austria) were employed to evaluate the mitochondrial respiratory function the at 37 °C. The assessment of permeabilized HaCaT and RPMI-7951 cells’ mitochondrial respiration was performed using a substrate uncoupler-inhibitor titration (SUIT) protocol, as previously described by Petruș et al. [61]. The cells were cultured in T75 culture flasks, washed with PBS, trypsinized, counted and resuspended (1 × 10^6^/mL) in mitochondrial respiration medium (MIRO5: MgCl_2_ 3 mM, EGTA 0.5 mM, taurine 20 mM, KH_2_PO_4_ 10 mM, K-lactobionate 60 mM, D-sucrose 110 mM, HEPES 20 mM and BSA 1 g/L, pH 7.1). The tested compounds GNP (10, 25 and 50 μΜ) and BA-GNP (10, 25 and 50 μΜ) were added immediately after the cells in the chambers of the device.

In order to obtain an extended profile of the cellular mitochondrial respiration, the membrane was permeabilized using a mild detergent: digitonin (35 μg/L × 10^6^ cells). The suitable digitonin concentration for selective plasma membrane permeabilization was determined in a previous respirometric protocol, as described by Pesta and Gnaiger [62]. The cells were first suspended in free media for 15 min. The mitochondrial respiration dependent on the endogenous ADP levels was the first respiratory rate recorded (Routine) after the steady-state was reached. The SUIT protocol subsequently applied consisted of the addition of the substrates, which allowed the measurement of the corresponding respiratory rates, as follows: (*i*) digitonin and the CI substrates (glutamate—G, 10 mM and malate—M, 5 mM)—LEAK respiratory rate (State2_CI_), (*ii*) ADP (5 mM)—active respiration dependent on complex I (CI) (OXPHOS_CI_), (*iii*) succinate—S (10 mM), a C II substrate—maximal OXPHOS capacity dependent on both CI and CII (OXPHOS_CI+CII_), (*iv*) cytochrome c (10 μM)—evaluation of mitochondrial membrane integrity (Cyt c), (*v*) oligomycin (1 μg/mL)—LEAK respiration dependent on CI and CII (State4_CI+II_), (*vi*) p-(trifluoromethoxy) phenylhydrazone carbonyl cyanide—FCCP (1 μM/step) successive titrations—the maximal respiratory capacity of the electron transport system (ETS_CI+II_), (*vii*) rotenone (0.5 μM)—maximal respiratory capacity of the electron transport system dependent solely on CI (ETS_CI_) and (*viii*) antimycin A (2.5 μM)—residual oxygen consumption (ROX). All obtained values were corrected after ROX.

All data were recorded using DatLab software (Oroboros Instruments GmbH, Innsbruck, Austria) and analyzed using GraphPad Prism 5 software (GraphPad Software, Inc., San Diego, CA, USA). Statistically significant differences were compared using two-way analysis of variance (ANOVA) with Bonferroni’s post hoc test. Values with *p* < 0.05 were considered to have a statistically significant difference (* *p* < 0.05, ** *p* < 0.01 and *** *p* < 0.01).

### 4.11. Western Blot

For Western blot analysis, HaCaT and RPMI-7951 (1 × 10^6^ cells) were stimulated with GNP (10, 25 and 50 μΜ) and BA-GNP (10, 25 and 50 μΜ) for 24 h at 37 °C. Following stimulation, the cells were lyzed in RIPA buffer (Thermo Fisher Scientific, Inc., Waltham, MA, USA) in the presence of Protease and Phospatase Inhibitor Cocktail (Thermo Fisher Scientific, Inc., Waltham, MA, USA). Protein concentration was determined using the Pierce™ Rapid Gold BCA Protein Assay Kit (Thermo Fisher Scientific, Inc., Waltham, MA, USA) and equal amounts of proteins (25 μg) were separated using Bolt™ 4–12% Bis-Tris Protein Gels (Mini Gel Tank-A25977, Thermo Fisher Scientific, Inc., Waltham, MA, USA). The resolved proteins were then transferred onto a nitrocellulose membrane by iBlot^®^ 2 Dry Blotting System (IB23001, Thermo Fisher Scientific, Inc., Waltham, MA, USA) and blocked with 5% skimmed milk. The blots were probed with anti-Bcl-2 Monoclonal Antibody (1 µg/mL) (MA5-11757) and anti-Bax Monoclonal Antibody (1:500 dilution, 33-6400) and detected by chemiluminescence using Goat anti-Mouse IgG (H+L) Superclonal™ Recombinant Secondary Antibody, HRP (1: 2000 dilution). GAPDH Polyclonal Antibody (1:1000 dilution, MA5-15738-D680) was used as a loading control. All antibodies were purchased from Thermo Fisher Scientific, Inc., Waltham, MA, USA. Chemiluminescent detection was performed using Pierce™ ECL Western Blotting Substrate (Thermo Fisher Scientific, Inc., Waltham, MA, USA) and the ChemiDoc MP Imaging System (170-8280) equipped with Image Lab image acquisition and analysis software v6.1 (BioRad, Hercules, CA, USA).

## 5. Conclusions

The current study reports the synthesis and physicochemical characterization of BA-GNP, followed by their biological assessment as anticancer agents against human melanoma. The conjugation of the phytocompound at the surface of metallic nanocarriers was mediated by cysteamine, which formed a covalent bond with the carboxylic group of betulinic acid. The synthesis resulted in spherical nanoparticles with diameters ranging from 14–20 nm, which were subsequently tested in HaCaT human keratinocytes and RPMI-7951 human melanoma cells, revealing selective cytotoxic properties and stronger antiproliferative effects compared to BA alone. Further studies indicated apoptotic activity as revealed by the morphological changes in melanoma cells and supported by Western blot results that showed the downregulation of the anti-apoptotic Bcl-2 expression combined with the upregulation of the pro-apoptotic Bax. The gold nanoconjugates were able to significantly inhibit mitochondrial respiration, thus further validating their mitocan activity. With regard to its conducive physicochemical properties, GNP remains a favorable platform for BA delivery. Additional surface modifications are also worth investigating for further tuning. However, further research is necessary to assess its potential for clinical applications.

## Figures and Tables

**Table 1:** Recorded hydrodynamic diameter, ζ potential and drug loading efficiency (DLE) for GNP and BA-GNP.

Sample	Hydrodynamic Diameter (nm)		Zeta Potential (mV)	DLE%
	Mean ± SD	PDI		
GNP	39 ± 3.4	0.15	−29.4 ± 0.9	-
BA-GNP	63 ± 5.7	0.23	−27.2 ± 0.7	31.6 ± 0.24

**Table 2:** Metallic content of GNP samples determined by X-ray fluorescence (XRF) analysis; only metals above 1000 ppm are shown.

Sample	Element (ppm)			
	Au	Si	Na	Other Metals
**GNP**	274,315	3945	5923	1248
**BA-GNP**	146,220	4213	3892	1145

**Table 3:** The comparative assessed irritant potential of BA, GNP and BA-GNP.

Tested Compounds and Controls	Irritation Score (IS)	Type of Effect
H_2_Od	0	Non-irritant
SLS	15.77	Irritant
GNP	0	Non-irritant
BA	0	Non-irritant
BA-GNP	0	Non-irritant

## Data Availability

Data is contained within the article.
